# Supplementary material for: Commensal Neisseria species share immune suppressive mechanisms with Neisseria gonorrhoeae
Source: PLoS One. 2023 Apr 7;18(4):e0284062. doi: 10.1371/journal.pone.0284062 (PMC10081783; doi:10.1371/journal.pone.0284062)
Supplement: S1 Fig — All of the PorB protein homologs except Ngo PorB have the 28 amino acid HTb tag at their N-terminus (designated HTb-PorB). The lanes are (left to right) Page Ruler Plus MW standards; Ngo PorB, in which the N-terminal HTb linker was cleaved off with TEV protease; HTb-Nmu PorB; HTb-Nla PorB; HTb-Nci PorB; and HTb-mouse Voltage Dependent Anion Channel (mVDAC). All of these proteins were refolded, purified on a Ni2+-NTA column, and then run on an S-300 gel filtration column. The proteins were from the pooled fractions from gel filtration. X, lane not included in Fig 1E. The gel was dried and scanned as a PDF image. (PDF) [file pone.0284062.s001.pdf]

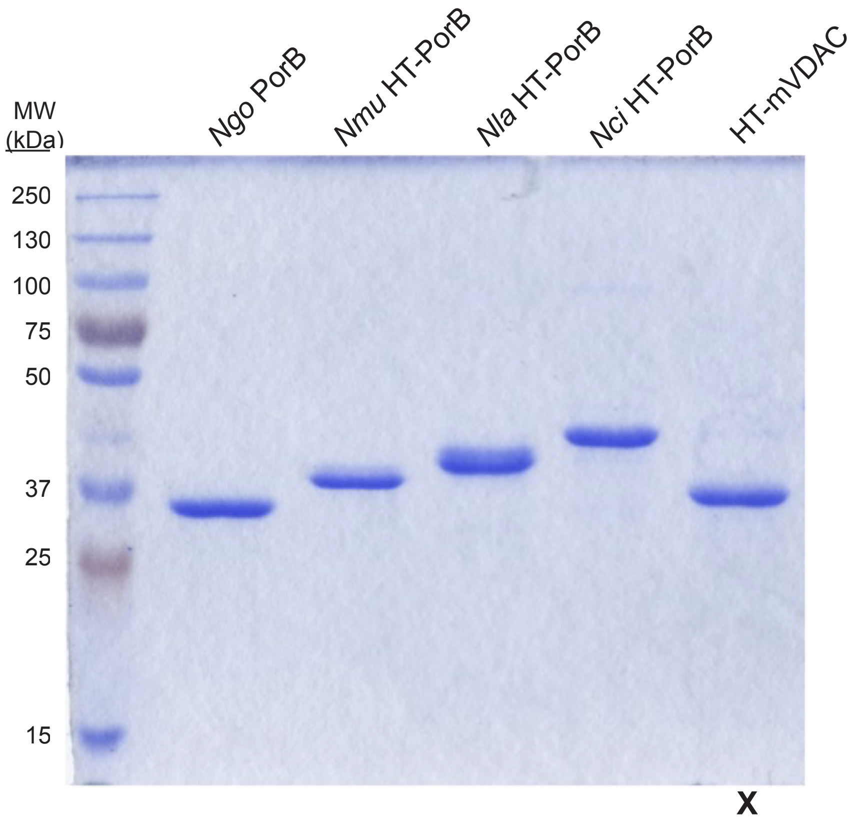

S1 Fig. Full gel of Fig 1E. All of the PorB protein homologs except *Ngo* PorB have the 28 amino acid HTb tag at their N-terminus (designated HTb-PorB). The lanes are (left to right) Page Ruler Plus MW standards; *Ngo* PorB, in which the N-terminal HTb linker was cleaved off with TEV protease; HTb-*Nmu* PorB; HTb-*Nla* PorB; HTb-*Nci* PorB; and HTb-mouse Voltage Dependent Anion Channel (mVDAC). All of these proteins were refolded, purified on a Ni^{2+}-NTA column, and then run on an S-300 gel filtration column. The proteins were from the pooled fractions from gel filtration. X, lane not included in Fig 1E.
