# Supplementary material for: Commensal Neisseria species share immune suppressive mechanisms with Neisseria gonorrhoeae
Source: PLoS One. 2023 Apr 7;18(4):e0284062. doi: 10.1371/journal.pone.0284062 (PMC10081783; doi:10.1371/journal.pone.0284062)
Supplement: S2 Fig — Refolding and purification of HT-PorB from N. mucosa expressed as inclusion bodies (IBs) in E. coli. The lanes are (left to right) Page Ruler Plus MW standards; Inclusion bodies of HT-Nmu PorB; Refolded Nmu HT-PorB; Filtered refolded Nmu HT-PorB; 15 mM imidazole bump from Ni2+-NTA column; refolded Ngo HT-PorB purified on a Ni2+-NTA column followed by gel filtration on an S-300 column; 1, 2, and 3 μL of the peak pooled fractions of Nmu HT-PorB following imidazole elution from the Ni2+-NTA column. Note the slight size increase in Ngo HT-PorB in this gel compared to TEV-cleaved Ngo PorB in S1 Fig. The HT tag has no effect on the capacity of Ngo PorB to inhibit DC-mediated T cell proliferation (Ref [14]). X, lane not included in Fig 1F. The gel was dried and scanned as a PDF image. (PDF) [file pone.0284062.s002.pdf]

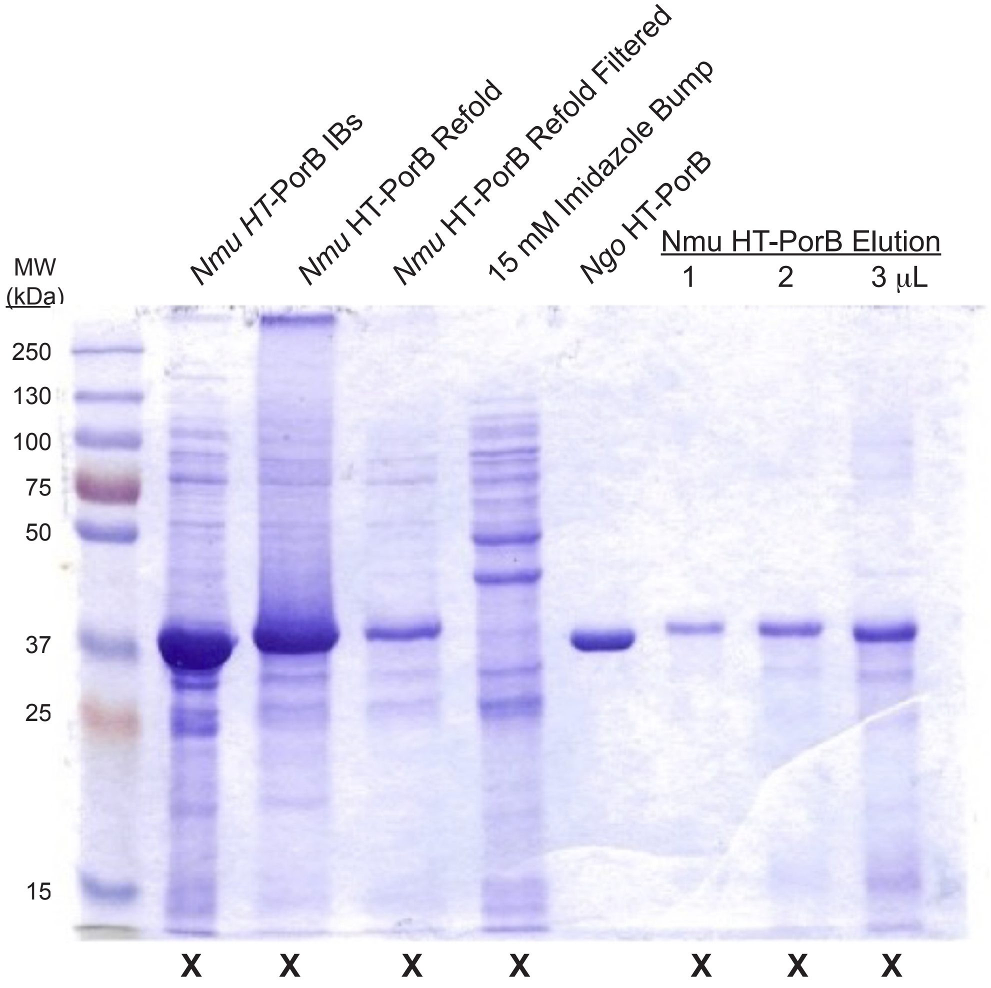

S2 Fig. Full gel of Fig 1F. Refolding and purification of HT-PorB from *N. mucosa* expressed as inclusion bodies (IBs) in *E. coli*. The lanes are (left to right) Page Ruler Plus MW standards; Inclusion bodies of HT-*Nmu* PorB; Refolded *Nmu* HT-PorB; Filtered refolded *Nmu* HT-PorB; 15 mM imidazole bump from Ni^{2+}-NTA column; refolded Ngo HT-PorB purified on a Ni^{2+}-NTA column followed by gel filtration on an S-300 column; 1, 2, and 3 μL of the peak pooled fractions of *Nmu* HT-PorB following imidazole elution from the Ni^{2+}-NTA column. Note the slight size increase in *Ngo* HT-PorB in this gel compared to TEV-cleaved *Ngo* PorB in Supplemental Fig 1A. The HT tag has no effect on the capacity of *Ngo* PorB to inhibit DC-mediated T cell proliferation (Ref 14). X, lane not included in Fig 1F.
