# Supplementary material for: Commensal Neisseria species share immune suppressive mechanisms with Neisseria gonorrhoeae
Source: PLoS One. 2023 Apr 7;18(4):e0284062. doi: 10.1371/journal.pone.0284062 (PMC10081783; doi:10.1371/journal.pone.0284062)
Supplement: S3 Fig — Conditioned medium prepared from cultures of commensal Neisseria species submitted to SDS-PAGE. Conditioned medium from cultures of the four Neisseria species was collected, and the number of particles (i.e. OMVs) was determined as described in Materials and Methods. Equal numbers of particles (left 4 lanes) or equal protein (right 4 lanes) were mixed with SDS-PAGE loading buffer and submitted to electrophoresis. The gel was stained with Sypro Ruby and imaged on a ChemiDoc Touch Imager (BioRad). The arrows indicate the PorB band in each species as determined by mass spectrometry (see Materials and Methods for details). X, lane not included in Fig 3. (PDF) [file pone.0284062.s003.pdf]

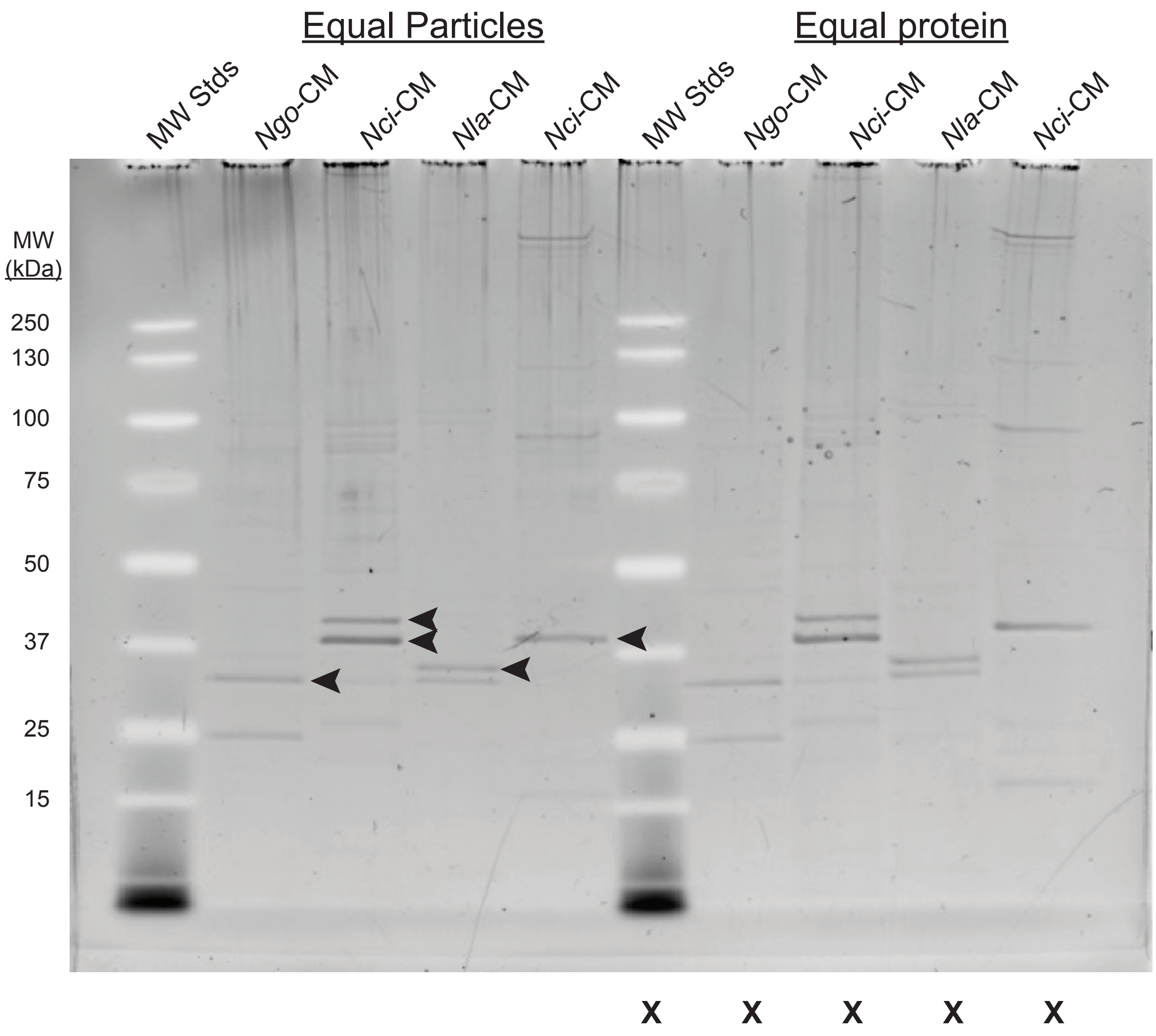

S3 Fig. Full gel from Fig 3. Conditioned medium prepared from cultures of commensal *Neisseria* species submitted to SDS-PAGE. Conditioned medium from cultures of the four *Neisseria* species was collected, and the number of particles (i.e. OMVs) was determined as described in Materials and Methods. Equal numbers of particles (left 4 lanes) or equal protein (right 4 lanes) were mixed with SDS-PAGE loading buffer and submitted to electrophoresis. The gel was stained with Sypro Ruby and imaged on a ChemiDoc Touch Imager (BioRad). The arrows indicate the PorB band in each species as determined by mass spectrometry (see Materials and Methods for details). X, lane not included in Fig 3.
