# Supplementary material for: Commensal Neisseria species share immune suppressive mechanisms with Neisseria gonorrhoeae
Source: PLoS One. 2023 Apr 7;18(4):e0284062. doi: 10.1371/journal.pone.0284062 (PMC10081783; doi:10.1371/journal.pone.0284062)
Supplement: Attachment — Submitted filename: Response to Review.docx [file pone.0284062.s004.docx]

Dear Dr. Al-Judaibi:

Thank you for your email on our initial submission [PONE-D-22-23059], with an editorial decision of minor revision. We have edited the manuscript to address the comments raised in the reviews and our changes are shown below for each point raised.

Reviewer #1:

- I don't have any criticism for this article. It is a well-designed study. Revealing the mechanisms of interaction between host and commensal bacteria may become more understandable with the contributions of such studies and may give an idea about why strains in the same bacterial genus show pathogenicity.
  I believe that this study, which reveals the immunosuppression mechanism caused by pathogenic and commensal Neisseria spp., will contribute to the scientific field.
  - We thank the reviewer for their kind comments on the manuscript.

Reviewer #2

- **Starting with title and abstract**, the title is proper for the substance of the paper. The abstract is brief the essential information of the paper. Although, in my opinion the author needs to add that to the abstract the research experiment was also done *in vitro.*
  - We have indicated that the results were done *in vitro* in the abstract (Line 30)
- **In terms of** **experimental technique**, the methods present in this manuscript clearly explain all required steps starting with Bacterial propagation and ending by the used Statistical analysis and it is a well-written. Also, all essential figures and tables were presented within paragraphs. Though, I would like the author to rename the first used figure in the line 120 page 7 to (**Fig 1 A-D**) instead of (**Fig 6** **A-D**) since the author started with it.
  - We have renumbered the figures and changed Fig 6 to Fig 1, and renumbered the remaining figures to accommodate this change.
- Rename **Fig 2b** to **Fig 2B** in the line 218 page 11.
  - We have made this change on Line 232, but this figure is now 3B instead of 2B
- In the figure 6 the author needs to add the used letters **(A-D & E)** in the paragraph represented in the page 13 to the figure.
  - We have added A through D for each of the gel filtration graphs, and E & F for the SDS-PAGE gels. Note that this figure is now **Fig 1**. We also include full gels in Supplemental Data.
- **In several places**, Author have used the bacterial name Neisseria instead of *Neisseria* with italic form (line 130 &line 141 page 7, line 228 page 11, line 258 page 13, line 282 page 14, line 309 page 15 and line 408 page 20).
  - We have gone through the manuscript and changed all occurrences of Neisseria to *Neisseria*
- In the last paragraph, it will be helpful if the author concluded the work findings and add the **Conclusions** part.
  - We have edited the text to make the last paragraph a conclusions paragraph.
- Finally, all references were listed by author under **References list** also recorded within paragraphs except **Ref. (32).**
  - Reference 32 (now reference 41) is cited in the Figure legend to new Fig 6. It cites the program used to generate the figure.
- In addition, I though it will be effective if the references updated to 2020, 2021 or 2022.
  - We have added several new references, some from 2020-2022. There is not a lot of new literature from 2020-2022 on immune suppression promoted by commensals, OMVs, or PorB.
- Overall though, this paper exceptional and it is an significant addition to the literature for immunity response by *Neisseria* species.
  - We thank the reviewer for the kind comments.

Sincerely,

|   Joseph A. Duncan, MD, PhD, FIDSA  Associate Professor of Medicine and Pharmacology  University of North Carolina School of Medicine  Department of Medicine, Division of Infectious Diseases  130 Mason Farm Road  Chapel Hill, NC 27599-7030  Phone: (919) 843-0715 Fax: (919) 843-1015 |   Robert A. Nicholas, PhD  Professor and Vice Chair of Pharmacology  University of North Carolina School of Medicine  120 Mason Farm Road  Chapel Hill, NC 27599-7365  Phone: (919) 966-6547 Fax: (919) 966-5640 |
| --- | --- |
