# Supplementary material for: Commensal Neisseria species share immune suppressive mechanisms with Neisseria gonorrhoeae
Source: PLoS One. 2023 Apr 7;18(4):e0284062. doi: 10.1371/journal.pone.0284062 (PMC10081783; doi:10.1371/journal.pone.0284062)
Supplement: Attachment — Submitted filename: Response to Review_v2.docx [file pone.0284062.s005.docx]

Dear Dr. Radcliff:

Thank you for your email on our submission [PONE-D-23-03331], with an editorial decision of minor revision. We have edited the manuscript to address the comments raised in the reviews, and our changes are shown below for each point raised.

Point 1:

- **Methods (line 152-153): were the DCs pulsed with OVA before combination with OT-II CD4+ T cells? Indicate source of OVA, concentration used/incubation time.**
  - We agree that the methods as previously written were not clear on these points. The DCs were pulsed for 24 hr with OVA in the presence of either live bacteria, conditioned medium, or purified PorB proteins. The cells were then washed to remove these and then incubated with the OT-II CD4+ T cells for 7 days. We have clarified this point in the revised manuscript and included all the requested information.

Point 2:

- **Methods (line 177-178): What ratio of DC:CD4+ responder cells was used for co-culture assays?**
  - The ratio of DC:CD4+ responder cells was 1:10 in the co-culture assays. We have included additional information to clarify the methods used.

Point 3:

- **Line 323: this sentence requires modification as it implies that reference number 37 includes a Gonorrhoea challenge study.**
- We thank the reviewer for catching this mis-statement; the study indeed did not include an *Ng* challenge study, and instead was looking at the cross-reactive bactericidal Abs produced by immunized mice. We have modified the original sentence to reflect this.

I am also including our response to a subsequent email to address two additional points before the article will be considered for publication. The points of the email are below, along with our responses.

- **To comply with PLOS ONE submissions requirements, in your Methods section, please provide additional information regarding the experiments involving animals and ensure you have included details on (1) methods of sacrifice, (2) methods of anesthesia and/or analgesia, and (3) efforts to alleviate suffering.**
- We have included additional information regarding the points above in the Ethics statement at the beginning of the Methods section.
- **2. Please note that PLOS ONE requires submissions reporting blot or gel data to comply in full with the reporting requirements described at** [**https://journals.plos.org/plosone/s/figures#loc-blot-and-gel-reporting-requirements**](https://journals.plos.org/plosone/s/figures#loc-blot-and-gel-reporting-requirements)**. We now require authors to provide the original unadjusted and uncropped images for any blot or gel data reported in PLOS ONE submissions. In our internal checks for your submission, we noted that you did not provide original raw image files supporting blot/gel data in response to our previous request.**
- Please note that we already provided the requested raw images in the Supplemental Information. However, it was apparently not named as requested (S1_raw_images). Therefore, we remade the document in Adobe Illustrator and renamed it S1_raw_images.pdf.

The revised manuscript (both tracked and clean) containing all of the above changes have now been uploaded. I have also removed Supplemental Information (since that file is now redundant with the new file S1_raw_images.pdf) and uploaded S1_raw_images.pdf in its place.

Sincerely,

|   Joseph A. Duncan, MD, PhD, FIDSA  Associate Professor of Medicine and Pharmacology  University of North Carolina School of Medicine  Department of Medicine, Division of Infectious Diseases  130 Mason Farm Road  Chapel Hill, NC 27599-7030  Phone: (919) 843-0715 Fax: (919) 843-1015 |   Robert A. Nicholas, PhD  Professor and Vice Chair of Pharmacology  University of North Carolina School of Medicine  120 Mason Farm Road  Chapel Hill, NC 27599-7365  Phone: (919) 966-6547 Fax: (919) 966-5640 |
| --- | --- |
